# Antibiotics and Airline Emergency Medical Kits

**DOI:** 10.3201/eid0906.030097

**Published:** 2003-06

**Authors:** Benjamin Bar-Oz, Bernadette Loughran

**Affiliations:** * Hadassah Medical Center, Jerusalem, Israel

**To the Editor:** Medical events during airline flights have drawn some attention in recently published articles and letters (*1*–*4*). We would like to share our experience of meningococcemia and meningococcal meningitis during a transatlantic flight.

In June 2000, a 20-year-old student with a mild viral illness (diagnosed before the flight) boarded a flight from Tel-Aviv, Israel, to Newark, New-Jersey, USA (approximate flight time, 11–12 hours), with a tour group of college-age students and their chaperones**.** We, a neonatologist and a neonatal intensive care nurse, were on the same flight to later transport a prematurely born infant from the United States back to Israel.

About 90 minutes before landing in New Jersey, the chief flight attendant asked me (B.B-O.) to check the passenger, who said he did not feel well. His medical history indicated no past illness, which was corroborated by the director of the tour group. The patient reported general malaise and numbness in his feet. In the 2 weeks before the flight, he had traveled in Israel, visiting cities, caves, and mountains. He and his group had slept in different hostels in those areas.

On examination, he was fully conscious, and his blood pressure and pulse rate were normal. He had a blue-purple skin rash, particularly on the upper extremities. The rash worsened in the course of 20 minutes and resembled the “blueberry muffin–like” rash described in other pathologic conditions. Considering a diagnosis of either tick-borne or meningococcal disease, I decided to give the patent the first dose of antibiotics after obtaining his verbal consent and consent from the head of the group. I also asked the crew to have an ambulance and a physician waiting for us at the destination airport.

When we checked the emergency medical kit, we found that it did not contain any antibiotics. For our transport mission, we had two ampules of cefotaxime, 2 g each, one of which we gave the patient. After we landed, an ambulance crew (which did not include a physician) took the patient to the nearest hospital. The patient died 2 hours later in the hospital emergency department. His laboratory tests showed meningococcal meningitis and meningococcemia. The Centers for Disease Control (CDC), the airline company, and Israel’s Ministry of Health notified all close contacts of the patient in Israel and during the transatlantic flight, including everyone in the tour group, and recommended that they be given chemoprophylaxis.

CDC has received 21 reports about air travel–associated meningococcal disease in 2 years; in 5 reports, the symptoms began before the plane arrived at its destination (*5*). However, advance notice of the symptoms was given only in our case. Although one case is not enough to substantiate recommendations, we believe that the appropriate authorities should require airline companies to add a broad-spectrum antibiotic preparation to the emergency kit. This drug should be used only when aircraft diversion is not possible and when the diagnosis is clinically identified or highly suspected.

We still wonder whether an earlier intervention and treatment with a more appropriate on-board antibiotic would have saved this young man.
